# Stepwise surgery with variable adjustments for severe blepharochalasis with multiple chemical sensitivity: a case report

**DOI:** 10.1080/23320885.2020.1719108

**Published:** 2020-01-29

**Authors:** Hisato Nagano, Takashi Domoto, Ryuichi Azuma, Tomoharu Kiyosawa

**Affiliations:** Department of Plastic and Reconstructive Surgery, National Defense Medical College Hospital, Tokorozawa, Japan

**Keywords:** Blepharochalasis, multiple chemical sensitivity, immunoglobulin A, immunological abnormality

## Abstract

We report a 29-year-old man with blepharochalasis and multiple chemical sensitivity (MCS). Although standard blepharoplasty with aponeurotic fixation was performed, palpebral ptosis recurred after 3 months. Eyelid function and appearance improved after additional operations. A stepwise surgical approach is appropriate in patients with blepharochalasis and MCS.

## Introduction

Blepharochalasis is a rare condition in which the eyelid skin becomes loose and redundant after repeated episodes of painless eyelid edema, which generally lasts for several days. It typically begins in childhood at an average age of 11 years and usually becomes less frequent in adulthood [1]. Many possible causes of blepharochalasis have been suggested, but the etiology remains unknown [1,2]. Several studies have suggested the possible involvement of immunological abnormalities [3–5]. We report a patient who had blepharochalasis and multiple chemical sensitivity (MCS), a condition in which low-level exposure to chemicals is associated with various systemic symptoms [6]. The patient provided written consent for publication. MCS is considered to differ from simple allergic disorders but is highly prevalent in patients with allergic diseases, such as asthma and atopic dermatitis [7].

## Case presentation

**Figure 1.**
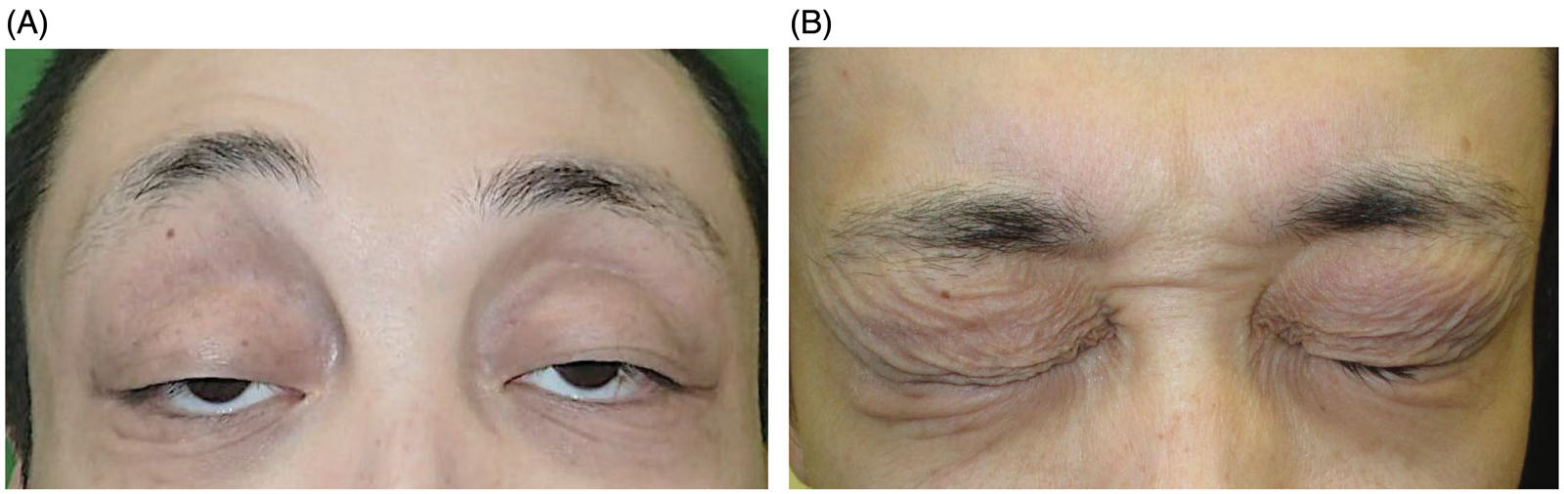
(A, B) Preoperative appearance: marked ptosis and crepey eyelid skin.

A 29-year-old man presented with bilateral ptosis associated with redundant, thin, crepey eyelid skin and hyperpigmentation (Figure 1(A,B)). He initially developed eyelid edema at the age of 10 years along with low-grade fever, headache, pharyngeal pain, abdominal pain and malaise. These symptoms recurred several times during the next decade but became rare after he reached adulthood. No apparent cause was found despite consulting several hospitals. He also developed severe MCS around the same time.

On physical exam, the MRD-1 (margin reflex distance-1: the distance between the upper lid margin and the center of the pupil) was −2.0 mm on the right and −1.5 mm on the left. He had fair levator function at 5 mm bilaterally. Many wrinkles were present on the lower eyelid, and the conjunctiva was exposed due to the ectropion. The snapback test was prolonged at 2 s. Laboratory tests were normal, including thyroid hormone levels.

**Figure 2.**
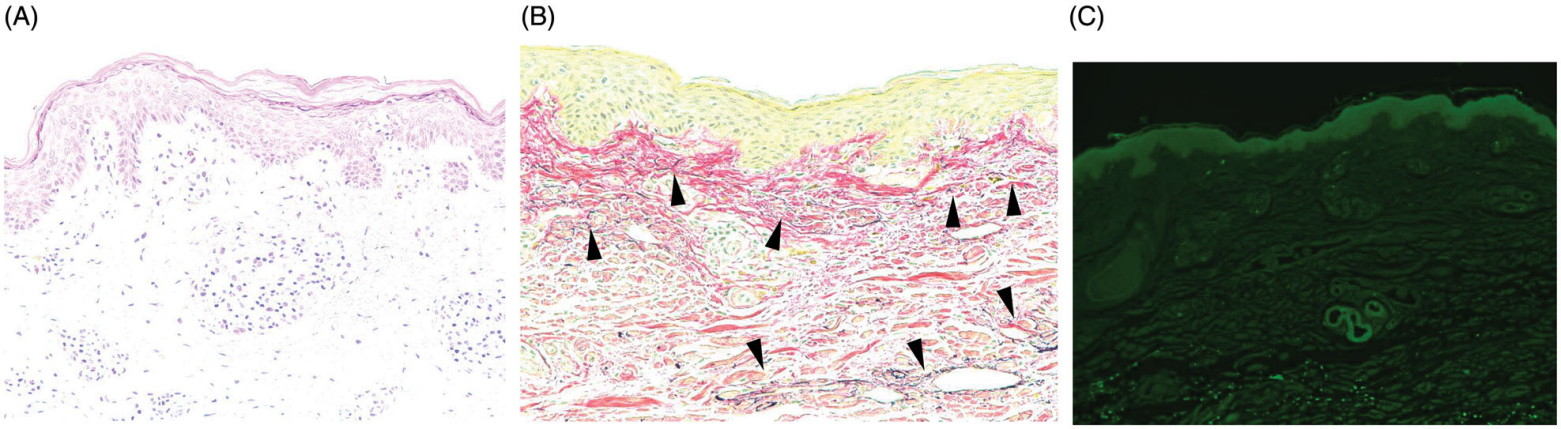
(A) Palpebral skin exhibits epidermal spongiosis and perivascular lymphocyte infiltration, suggesting superficial dermatitis compatible with blepharochalasis (hematoxylin and eosin, ×200). (B) Elastic fibers are reduced in the superficial dermis and exhibit fragmentation (arrowhead, elastica van Gieson, ×200). (C) Immunostaining reveals perivascular IgA deposits (arrow, ×100).

Surgery was conducted under local anesthesia to improve ptosis. The upper eyelid skin was excised bilaterally (maximum width: 13 mm). The levator muscle was found to be elongated. Standard blepharoplasty with aponeurotic fixation was performed. The levator aponeurosis was advanced by 5 mm and sutured with 7-0 nylon at three points 2 mm caudal to the superior edge of the tarsus. Specimens of upper eyelid skin, orbicularis oculi muscle, orbital septum and fat were submitted for histological examination, which revealed edema of the dermis and perivascular infiltration of lymphocytes (Figure 2(A)). Abnormalities of the orbicularis oculi, orbital septum and fat were less marked. Elastica van Gieson staining exhibited a decrease of dermal elastic fibers (Figure 2(B)). Direct immunofluorescence revealed perivascular deposits of immunoglobulin A (IgA) in the dermis (Figure 2(C)). We diagnosed blepharochalasis based on the clinical features and histopathological findings.

**Figure 3.**
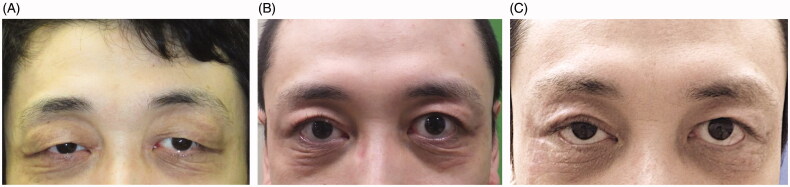
(A) Appearance at 3 months after the first operation. (B) Appearance at 8 months after the second operation. (C) Appearance at 15 months after the third operation.

Three months later, bilateral palpebral ptosis recurred (Figure 3(A)). After careful consideration, we performed a second operation. Additional upper eyelid skin (maximum width: 14 mm) was resected, and the levator aponeurosis was additionally advanced to the tarsus 2 mm from its previous position. Eight months after the second surgery, blepharoptosis was improved, but skin redundancy of the lower eyelid and ectropion remained (Figure 3(B)). We then performed the Kuhnt-Szymanowski procedure due to tightening of the lower eyelid and resected the redundant skin. Pentagonal wedge resections of the lateral posterior lamella (5 mm width) and the redundant lower eyelid skin (8 mm maximum width) were performed. At 15 months follow-up, the MRD-1 was +4.0 mm on the right and +5.0 mm on the left; there was no lower lid wrinkling, exposed conjunctiva or recurrence of palpebral edema (Figure 3(C)). The patient was satisfied with the esthetic result.

## Discussion

Blepharochalasis was first described by Beer and named by Fuchs [8] in 1817. Repeated episodes of eyelid edema cause stretching and damage to the soft tissues, including the palpebral skin, which becomes thin and flaccid with many fine wrinkles and appears bronze in color.

Our patient had severe blepharochalasis: both the upper and lower eyelids were affected bilaterally, and up to 27 mm of redundant eyelid skin was surgically removed. The severity may have been due to concomitant MCS. Although blepharochalasis associated with systemic symptoms has been previously reported, the cause remains unclear [1]. However, immunofluorescence studies have detected IgA antibodies on elastic fibers in blepharochalasis patients [3,4,9,10], suggesting an immunologic basis. In our patient, perivascular IgA deposits were detected in the dermis. Therefore, his blepharochalasis may have been related to MCS.

Treatment of blepharochalasis involves correcting the aponeurotic ptosis and resecting the redundant palpebral skin [11], and surgery should be performed during the quiescent phase [11–13]. Since surgical overcorrection is frequently associated with complications, Koursh et al. [1] and Bergin et al. [12] have advocated intentional undercorrection. However, the appropriate extent of skin resection is difficult to estimate. In our patient, we initially resected the maximum amount of eyelid skin that still allowed complete eyelid closure; he was still able to close his eyes fully after surgery. However, palpebral ptosis recurred after 3 months, suggesting the need for further surgery. Huemer et al. and Takahashi et al. reported similar cases of recurrence after surgery [14,15]. Although the immediate postoperative result was satisfactory, their patients also required a second operation. A stepwise surgical approach seems to prevent complications and appears appropriate in managing blepharochalasis.
